# “Stable” vs. “silent progressive multiple sclerosis”: a real-world retrospective clinical imaging Brazilian study

**DOI:** 10.1590/0004-282X-ANP-2020-0234

**Published:** 2022-02-21

**Authors:** Gustavo Medeiros Andrade FIGUEIRA, Paula Vallegas SOARES, Raquel Custodio da SILVEIRA, Fernando Faria Andrade FIGUEIRA

**Affiliations:** 1 Hospital São Francisco na Providência de Deus, Departamento de Neurologia, Rio de Janeiro RJ, Brazil.

**Keywords:** Multiple Sclerosis, Neuroimaging, Corpus Callosum, Esclerose Múltipla, Neuroimagem, Corpo Caloso

## Abstract

**Background:**

Clinical and imaging are required to characterize activity and progression in MS. The parameters for activity are well defined but not those for progression. The ideal aim for long-term treatment is that neither clinical nor imaging signs of disease should be present, and also no brain atrophy.

**Objectives:**

To conduct a comparative clinical-imaging study focusing on MRI brain volumetry.

**Methods:**

174 consecutive relapsing-remitting MS patients (McDonald 2001) were studied, focusing on activity and progression. Annual clinical evaluations (relapse rate and EDSS) and MRI data, along with the annualized evolution of the corpus callosum index (CCI), were compared.

**Results:**

Out of 174 patients, 148 were considered clinically “stable” based on EDSS. However, 33 (22.2%) out of this group showed annualized reductions in CCI of more than 0.5%, which was the cutoff for defining significant brain atrophy.

**Conclusions:**

Among apparently “stable” relapsing-remitting MS patients, 1/5 showed significant brain atrophy over a follow-up period of at least 7 years. We consider it reasonable to suggest that MRI volume sequences should be included in follow-up protocols, so as to provide information on the real treatment response status.

## RESUMO

**Antecedentes:**

Critérios clínicos e de imagem são necessários para caracterizar atividade e progressão em esclerose múltipla (EM). Os parâmetros para a atividade são bem definidos, o que não ocorre com a progressão. O objetivo ideal para tratamento em longo prazo inclui ausência de sinais clínicos e de imagem, assim como inexistência de atrofia cerebral.

**Objetivos:**

Estudo comparativo de aspectos clínicos e correlatos de imagem, com foco em volumetria cerebral.

**Métodos:**

Foram avaliados 174 pacientes consecutivos com o diagnóstico de EM surto-remissiva (McDonald 2001), com foco em dados de atividade e progressão. A avaliação clínica anual (taxa de surtos e escala expandida do estado de incapacidade - EDSS) e dados de imagem, assim como a evolução anualizada do Índice de Corpo Caloso (CCI), foram comparados.

**Resultados:**

Da amostra inicial de 174 pacientes, 148 foram considerados “clinicamente estáveis” com base na EDSS. Todavia, 33 (22,2%) pacientes desse grupo mostraram redução volumétrica anualizada no índice de corpo caloso acima de 0,5%, nível de corte para definir a atrofia cerebral significativa.

**Conclusões:**

Entre pacientes de EM surto-remissiva aparentemente estáveis, cerca de 1/5 apresentou sinais de atrofia cerebral significativa em sete anos de seguimento. Consideramos razoável sugerir que sequências de volumetria deveriam ser incluídas nos protocolos de seguimento, fornecendo informação quanto ao real estado da resposta ao tratamento.

## INTRODUCTION

Relapses and remissions are clinical hallmarks of multiple sclerosis (MS) and were the basis for the original Lublin et al. classical phenotypes of the disease^1^. Since the 1990s, neurologists worldwide have recognized relapsing-remitting, primary and secondary progressive MS as the prototypes for classifying their patients using exclusively clinical evidence of relapses and measurements of progression, using scales such as John Kurtzke’s Expanded Disability Scale Score (EDSS)^2^.

**Figure 1.**
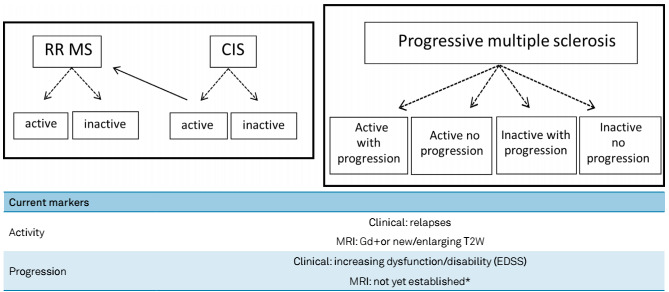
Clinical and imaging information on new phenotypes has been gathered, thus leading to earlier and more sensitive characterization of activity and progression in multiple sclerosis patients.

However, new phenotypes were required. The development of new drugs has created more aggressive options for use as early as possible on non-responders or therapeutic failures. These new options, in association with the inclusion of imaging data to demonstrate active lesions, even if asymptomatic, have brought a new approach. Clinical and imaging information about these phenotypes has been gathered (Figure 1), and this has led to earlier and more sensitive characterization of activity and progression in MS patients^3^.

*The following are under consideration: increasing number and volume of T1W hypointense lesions, brain volume loss, magnetization transfer ratio (MTR) and diffusion tensor imaging (DTI).

Clinical relapses and their magnetic resonance imaging (MRI) correlates are well-defined and accepted parameters for demonstrating the inflammatory activity in MS. On the other hand, the progressive component of the disease is a more subtle and insidious process, for which clear-cut, practical and sensitive markers are still far from being reached.

The aim of this study was to analyze the impact of a practical imaging method for measuring axonal loss and corpus callosum atrophy among a real-world sample of apparently clinically stable MS patients, and its implications for clinical practice.

## METHODS

We included patients from retrospectively analyzed files on 185 consecutive non-selected cases from our program for treating patients with diagnoses of relapsing-remitting MS (in accordance with the McDonald 2001 criteria). These patients were seen between 2001 and 2012; they were all on regular treatment, with self-reported full adherence. At least three MRI studies were available for each patient, all analyzed by the same observer (F.F.A.F.), at the baseline, an intermediate time (at variable times) and at the end of the follow-up period, with a proper protocol, which thus led to reliable evaluation of activity and progression over at least a 7-year period.

Conventional MRI studies were acquired using a Siemens 1.5T scan device, in slices of 3 mm with no gap. The scans consisted of at least an axial T1W pre and post-gadolinium injection, axial T2W/FLAIR and sagittal T1W, among other sequences, according to the indications, which were established in case-by-case evaluations.

All the patients gave their informed consent, and the study protocol was approved by the Ethics and Humanity Committee of our institution. Eleven files were excluded: 3 cases due to lost follow-up and 8 due to insufficient data, thus resulting in a study group of 174 patients. As a real-world sample, both routine clinical and imaging evaluations were performed by the same two observers (G.M.A.F. and F.F.A.F.), which was a possible limitation of our study. The evaluations included the relapse rate and EDSS, measured at least annually, for no less than 7 years (mean, 8.4). All patients with no evidence of incapacity progression over this period, based on an increase in EDSS of 0.5 points or more, were considered “clinically stable”. MRI activity data included the presence of gadolinium-positive lesions or new/enlarging T2W lesions, as originally defined by Barkhof and Tintoré^4^ ^,^ ^5^ and adopted by McDonald and the International Panel^6^. Corpus callosum atrophy was evaluated in terms of the annualized evolution of the corpus callosum index (CCI), as previously described by our group^7^.

On the other hand, we determined the burden of disease by manually measuring T2W lesion counts on axial FLAIR sequences, and this was significantly greater in “progressive” patients (8.7 vs. 5.2; p=0.001, Fisher). For methodological reasons, we did not take into account enlarging lesions.

## RESULTS

**Figure 2.**
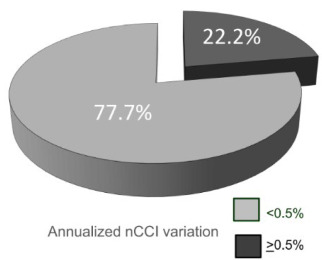
In this “stable” group, 33/148 (22.2%) showed an annualized reduction in CCI of more than 0.5%.

The demographic data matched with the population of our treatment program. After a 7-year follow-up, 148 out of 174 showed no evidence of progression on EDSS and were considered “clinically stable” (Table 1). Nevertheless, in this “stable” group, 33/148 (22.2%) showed an annualized reduction in CCI of more than 0.5% (Figure 2), a score that our original study showed to be the cutoff for distinguishing a significant loss of brain volume, compared with the controls^7^. As expected, the “progressive” patients were older (37.3 vs. 32.4 years old), had had more time since the disease diagnosis (8.7 vs. 6.6 years), higher disability scores on EDSS (3.9 vs. 3.1) and higher annualized relapse rates (0.22 vs. 0.18) than the “stable” ones, but none of these variables were statistically significant (Table 2). For methodological reasons, we did not take into account enlarging lesions.

**Table 1.** After a 7-year follow-up, 148 out of 174 patients showed no evidence of progression in Expanded Disability Scale Score and were considered “clinically stable”

“Clinically stable” group characteristics	
N	148
Mean age (range)	36.6 (17-61)
Male/Female	62/86
Years of disease (mean)	8.4 (3.7-11.7)
Mean EDSS (range)	3.7 (1-5.5)
ARR	0.21
Mean T2W lesions (range)	7.3 (4-17)
Annualized nCCI (range)	0.331 (0.28-0.583)

EDSS: Expanded Disability Scale Score; ARR: Annualized Relapse Rate; T2W: Magnetic Resonance Imaging T2-weighted image; nCCI: normalized corpus callosum index.

**Table 2.** Considering the normalized corpus callosum index cutoff of 0.5%, a subgroup of the “clinically stable” patients seemed to behave as “progressive”

	“Stable” patients	“Progressive” patients
N	115 (77.7%)	33 (22.2%)
Mean age (range)	32.4 (17-44)	37.3 (27-61)
Male/Female	44/71	18/15
Years of disease (mean)	6.3 (3.7-8.8)	8.6 (7.1-11.7)
Mean EDSS (range)	3.1 (1-4)	3.9 (2.5-5-5)
ARR	0.18	0.22
Mean T2W lesions (range)	5.2 (4-9)	8.7 (6-17)
Annualized nCCI (range)	0.317 (0.28-0.433)	0.541 (0.508-0.583)

EDSS: Expanded Disability Scale Score; ARR: annualized relapse rate; T2W: Magnetic Resonance Imaging T2-weighted image; nCCI: normalized corpus callosum index.

Also of note, the gender prevalence ratio of the “progressive” group showed a shift compared with the “stable” ones and to the whole sample (female 45.5% vs. 61.7% and 58.1% respectively; Table 1). These are interesting data to be studied, but far beyond the scope of this paper.

## DISCUSSION

In a pivotal study on monoclonal antibodies, it was proposed that the ideal aim for long-term optimal treatment would be to reach an absence of clinical and MRI correlates of acute relapses, together with no progression of disability, i.e. the so-called “no evidence of disease activity” or NEDA 3^8^. This concept was stringently enriched through inclusion of volumetric data showing absence of brain atrophy on MRI longitudinal studies, defined as the so-called NEDA 4^9^. Moreover, recent data have suggested that axonal loss may be clinically “silent”, in spite of unequivocal imaging evidence^10^. Nevertheless, imaging methodologies for measuring axonal loss and brain atrophy still demand validation, use non-conventional MRI sequences and require expertise that is not always available in most treatment centers.

The current criteria for phenotypes of evolution in MS define *worsening* disease as increasing disability due to disease activity, both from clinical and/or imaging data. These are clear and useful markers, especially for optimal treatment follow-up. Otherwise, *progression* refers to an increase in disability that is not related to relapses or active lesions on MRI. Progression might be related to a degenerative component of the disease, hallmarked by axonal loss and brain atrophy, but which is not always clinically apparent^3^. While activity is a concept that is easy to determine in daily practice, progression is not at all. In spite of the widespread use of clinical standards of evaluation, such as EDSS and MSFC, these lack enough sensitivity to be used as a parameter for progressive disease, especially over the short term. Cognitive batteries are usually complex and time consuming^11^. Similarly, imaging markers of axonal loss that use non-conventional sequences require expertise for their interpretation, which is not always available in most treatment centers^12^ ^,^ ^13^.

**Figure 3.**
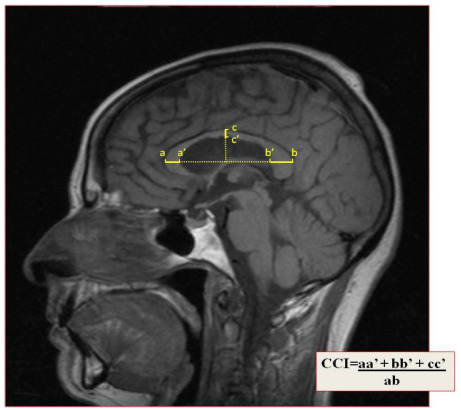
The corpus callosum index is a simple and feasible tool obtained from two-dimensional measurement of the corpus callosum using an orthogonal semi-automated linear model, applied to a conventional mid-sagittal T1-Weighted Magnetic Resonance Imaging sequence.

The corpus callosum is the largest axonal interhemispheric brain connection. It seems reasonable to infer that diffuse axonal loss may be expressed through its morphological changes. The CCI is a simple and feasible index that is obtained from two-dimensional measurement of the corpus callosum using an orthogonal semi-automated linear model applied to a conventional mid-sagittal T1W MRI sequence (Figure 3). It was recently replicated in several centers and a normalized CCI was shown to be a reliable marker for brain atrophy, with good intra and inter-observer ratings, and with correlations with brain parenchymal fraction, EDSS and the speed of information processing measured through the Paced Auditory Serial Addiction Test (PASAT)^7^ ^,^ ^14^ ^,^ ^15^ ^,^ ^16^. Compared with a blinded radiologist, CCI determination showed interobserver disagreement of 0.92% (SD=0.32; p=0.003)^17^.

In this study, “progressive” patients had higher lesion counts on T2W/FLAIR sequences at baseline than “stable” ones, thus suggesting that more active disease should be a predictive factor for axonal loss and callosal atrophy in late stages^13^. Also for methodological purposes, we did not take into account infratentorial and spinal cord lesions, which are both relevant to disability but were beyond the scope of our paper.

Burden of disease, as expressed through higher T2W hyperintense lesion counts, is not an easy parameter for clinical practice, but in our sample high scores correlated with more clinically active disease and more axonal loss. Its correlation with CCI points towards a reasonable association between aggressiveness of disease and progression.

MS is a multifaceted disease: inflammation and neurodegeneration evolve together. Over recent years, use of MRI has dramatically changed the approaches to MS. Imaging technologies are continuing to emerge, with improvements in diagnostic sensitivity and specificity and optimization of follow-up, and these are also providing new information on the pathophysiology of this disease^17^. They are welcome, but still far from being available in most centers.

In this real-world proof-of-concept study, we randomly enrolled patients who were in a regular program of treatment, and these patients were followed for 7 year. Data were collected using conventional daily-practice methodology for diagnosis and follow-up.

Among 148 apparently “clinically stable” MS patients on regular treatment schedules and fulfilling the criteria for NEDA-3 over a period of at least 7 years, more than 20% had significant progressive callosal atrophy. Thus, less than 80% achieved the criteria for NEDA-4, which therefore raises questions regarding the optimal treatment response: is NEDA-3 enough? Or should cell loss and brain atrophy be a target, even if “clinically silent”?

### Limitations and strengths

Our patients were enrolled in a real-world scenario: using serial clinical examination as well as conventional imaging analysis, always by the same staff observers (G.M.A.F and F.F.A.F), as part of a regular program of treatment in our hospital. This can be considered to be a weakness in our study, academically, but it reflects the daily-practice approach.

In spite of the low number of patients enrolled, it seems reasonable to conclude that a regular and practical brain volumetry technique can provide valuable information about the real state of the treatment response. In this manner, these “silent progressive” patients that are candidates for a switch to more active therapeutic strategies can be selected. Given that this was a proof-of-concept study, our data need to be replicated by other Centers, maybe with more robust numbers of patients.
